# Metal-free electrocatalytic hydrogen oxidation using frustrated Lewis pairs and carbon-based Lewis acids†
†Electronic supplementary information (ESI) available. See DOI: 10.1039/c5sc04564a


**DOI:** 10.1039/c5sc04564a

**Published:** 2016-01-06

**Authors:** Elliot J. Lawrence, Ewan R. Clark, Liam D. Curless, James M. Courtney, Robin J. Blagg, Michael J. Ingleson, Gregory G. Wildgoose

**Affiliations:** a Energy & Materials Laboratory , School of Chemistry , University of East Anglia , Norwich Research Park , Norwich , NR4 7TJ , UK . Email: G.Wildgoose@uea.ac.uk; b School of Chemistry , University of Manchester , Oxford Road , Manchester M13 9PL , UK . Email: Michael.Ingleson@manchester.ac.uk

## Abstract

Whilst hydrogen is a potentially clean fuel for energy storage and utilisation technologies, its conversion to electricity comes at a high energetic cost. This demands the use of rare and expensive precious metal electrocatalysts. Electrochemical-frustrated Lewis pairs offer a metal-free, CO tolerant pathway to the electrocatalysis of hydrogen oxidation. They function by combining the hydrogen-activating ability of frustrated Lewis pairs (FLPs) with electrochemical oxidation of the resultant hydride. Here we present an electrochemical–FLP approach that utilises two different Lewis acids – a carbon-based *N*-methylacridinium cation that possesses excellent electrochemical attributes, and a borane that exhibits fast hydrogen cleavage kinetics and functions as a “hydride shuttle”. This synergistic interaction provides a system that is electrocatalytic with respect to the carbon-based Lewis acid, decreases the required potential for hydrogen oxidation by 1 V, and can be recycled multiple times.

## Abstract 2

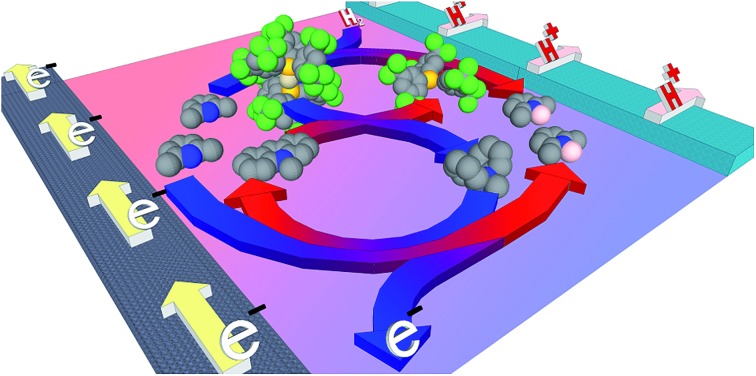
The synergistic interaction of a carbon-centred Lewis acid and borane “hydride shuttle” offers a metal-free, CO tolerant pathway to hydrogen oxidation.

## Introduction

As the demand for sustainable and carbon-neutral sources of electricity increases, there is a need for new technologies that allow the efficient storage and utilization of energy.^1^ H_2_ is attractive as an energy vector since energy from renewable sources may be stored in its chemical bond, and then cleanly and safely released as electricity using fuel cell technology.^2^

Unfortunately, in the absence of a suitable electrocatalyst, the conversion of H_2_ into two protons and two electrons is slow and must be driven by a large overpotential (voltage). Precious metal electrodes (such as Pt) provide an electrocatalytic effect that is indicated by a marked increase in current and a shift in the electrode reaction to a lower potential (voltage).^3^,^4^ However, the high cost and low abundance of such materials presents a significant barrier to the wide-spread adoption of current H_2_ fuel cell technology. There is clearly a need to develop new H_2_ oxidation electrocatalysts that are free from precious metals. Progress has been made in this area using bioinspired catalysts^5^–^7^ that contain either Ni^8^–^10^ or Fe^11^–^13^ centres. However, a significant weakness of existing electrocatalysts (Pt and the majority of hydrogenase enzyme mimics) is that they are highly sensitive to CO binding and inhibition.^5^,^14^ Trace amounts of CO are inevitably present in H_2_ that is commercially produced from hydrocarbon feedstocks. Worse still, for indirect methanol fuel cells (a combined H_2_ fuel cell and MeOH reformer) a CO removal process is often necessary to prevent electrocatalyst poisoning.^15^

An alternative metal-free strategy uses frustrated Lewis pairs (FLPs) to activate H_2_. Since their discovery by Stephan's group in 2006,^16^ research involving FLPs has grown apace.^17^–^23^ FLPs, formed from the combination of suitably sterically encumbered Lewis acids (LA) and bases, are precluded from forming classical Lewis adducts; such systems can heterolytically cleave H_2_ to generate hydridic and protic components. The hydrogenation of a wide range of functional groups including imines, enamines, nitriles,^24^–^27^ aldehydes,^28^ and ketones^29^–^33^ using FLPs has been reported.

**Fig. 1:**
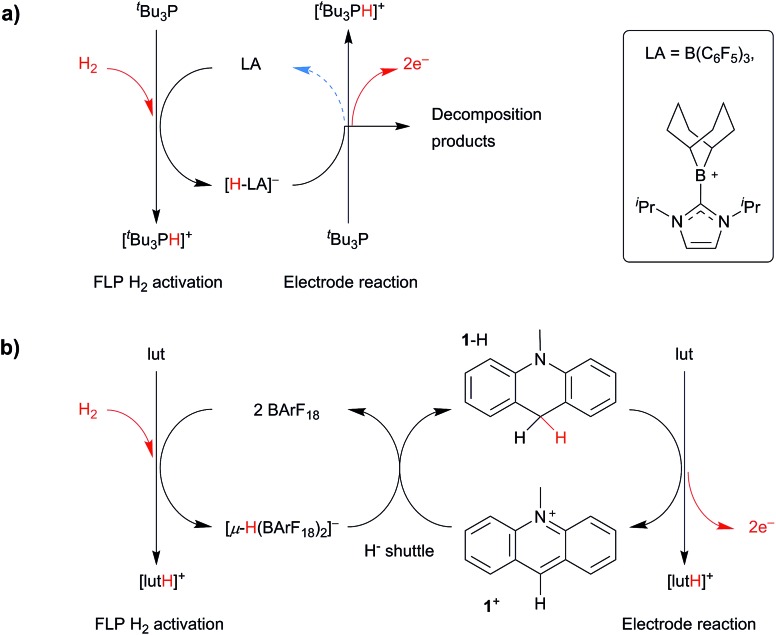
(a) The borane-only electrochemical–FLP system that was limited by the electrochemical stability of the borane, and (b) a carbon-based electrochemical–FLP that uses a redox inactive borane as a hydride shuttle. Note that any residual charges are counterbalanced by the supporting electrolyte [^*n*^Bu_4_N][B(C_6_F_5_)_4_], which is present in large excess.

In 2014, Wildgoose and Ashley pioneered a new metal-free route to H_2_ oxidation using a combined “electrochemical–FLP” approach.^34^,^35^ This enables the conversion of H_2_ into two protons and two electrons at cheap and ubiquitous carbon electrodes. Using the archetypal ^*t*^Bu_3_P/B(C_6_F_5_)_3_ system (Fig. 1a),^34^,^36^ the voltage (driving energy) required to oxidize H_2_ was decreased by 610 mV (*ca.* 118 kJ mol^–1^). Later, we applied this “electrochemical–FLP” approach to Stephan's NHC-stabilized borenium cation (Fig. 1a),^35^,^37^ which decreased the voltage required for H_2_ oxidation by 910 mV (*ca.* 176 kJ mol^–1^). However, a detailed mechanistic study of both these electrochemical–FLP systems revealed several limitations that significantly hindered their catalytic turnover, efficiency, and application as replacement electrocatalysts for energy applications. This included the side-reaction of radical intermediates with solvent/electrolyte during electrolysis, and the deactivation of electrocatalyst *via* its reaction with electrogenerated protons. Whilst the borenium cation offered an improvement over the borane system, the rate of H_2_ cleavage by this borenium–FLP is far too slow.

Whilst the majority of research involving FLP H_2_ activation has been focused on boron-centred Lewis acids, the Ingleson group have recently reported a FLP derived from salts of the *N*-methylacridinium cation (**1^+^**), a carbon-centred Lewis acid, and the Lewis base 2,6-lutidine (lut).^38^,^39^ **1^+^** is inexpensive, easy to synthesise, and is similar in structure to the NADH/NAD^+^ coenzyme system that is found in biological redox systems.^40^–^44^ Furthermore, in 1990, Savéant and co-workers elucidated all the pertinent non-aqueous mechanistic parameters of the **1^+^**/*N*-methylacridane (**1**-H) redox couple both in the presence and the absence of a Brønsted base.^43^,^44^ The oxidation of **1**-H involves an ECE-DISP1 mechanism and results in the net formation of two electrons and an electrogenerated proton (Scheme S1†). Compared to either of the boron-based electrochemical–FLP systems reported previously, the standard potential of the **1**-H/[**1**-H]˙^+^ couple is relatively low (+0.48 ± 0.01 V *vs.* Cp_2_Fe^0/+^ in MeCN). Also, **1**-H is insufficiently hydridic to react with any electrogenerated H^+^ produced, so no competing H_2_ evolution reaction (the reverse reaction of H_2_ cleavage by the FLP) occurs. Together, these attributes (ease of synthesis, high hydride affinity of **1^+^**, favourable oxidation potential of **1**-H and the lack of side-reactions during electrolysis) combine to make the carbon-based **1**-H/**1^+^** system a highly attractive candidate for electrochemical–FLP studies. The only limitation of the **1^+^**/lut FLP is that the rate of H_2_ cleavage is very slow – requiring >9 days for almost complete H_2_ activation at 60 °C and 4 bar.^38^

Fortunately, a solution to this final problem is available to us. We have recently examined the possibility of using tris[3,5-bis(trifluoromethyl)phenyl]borane (BArF_18_) as the Lewis acidic component of an electrochemical–FLP system.^45^ The activation of H_2_ by BArF_18_-containing FLPs is rapid and favours the formation of the bridging hydride, [(μ-H) (BArF_18_)_2_]^–^.^46^,^47^ However, the oxidation potential of [(μ-H) (BArF_18_)_2_]^–^ is too positive to be useful for electrochemical–FLP applications (*ca.* +1.55 V *vs.* Cp_2_Fe^0/+^) and resembles that of molecular H_2_ – BArF_18_ is not electrocatalytic towards H_2_ oxidation.

In this paper we combine the rapid H_2_ cleavage kinetics of BArF_18_-derived FLPs with the stability and efficiency of the carbon-centred Lewis acid, **1^+^**. Using this approach, the bridging hydride, [(μ-H) (BArF_18_)_2_]^–^, effectively functions as a redox inactive “hydride shuttle” to generate **1**-H from **1^+^** (Fig. 1b). As we demonstrate herein, the “hydride shuttle” combines the rapid cleavage of H_2_ by the BArF_18_/lut FLP with the favourable electrochemical properties of **1**-H. This provides an improved electrocatalytic system, with numerous advantages over previous electrochemical–FLP systems: a *ca.* 1 V decrease in the voltage for H_2_ oxidation at a carbon electrode; a metal-free system that is catalytic in **1^+^**, turns over efficiently and can be recharged multiple times; no undesirable H_2_ evolution side-reactions and a marked improvement in FLP H_2_ cleavage kinetics compared to carbon-based Lewis acids alone. We also demonstrate that, in stark contrast to conventional H_2_ oxidation electrocatalysts, this electrochemical–FLP system is tolerant of CO.

## Results and discussion

### BArF_18_ as a hydride shuttle

Bridging borohydrides are generally considered to be less hydridic than their terminal analogues. However, NMR experiments show that [(μ-H) (BArF_18_)_2_]^–^ is capable of transferring hydride to B(C_6_F_5_)_3_. When a suspension of [tmpH][(μ-H) (BArF_18_)_2_] (tmp = 2,2,6,6-tetramethylpiperidine) in CD_2_Cl_2_ is treated with B(C_6_F_5_)_3_, the formation of a clear, colourless solution is observed, indicating that the sparingly soluble starting material has undergone reaction. Indeed, ^1^H, ^19^F{^1^H} and ^11^B NMR spectra of the reaction mixture indicate the formation of [tmpH][HB(C_6_F_5_)_3_] and two equivalents of BArF_18_ (Fig. S1–S3†).^28^,^46^,^47^ The sequestration of hydride by B(C_6_F_5_)_3_ likely reflects the greater electrophilicity of B(C_6_F_5_)_3_ (*E*° = –1.52 V *vs.* Cp_2_Fe^0/+^) compared to BArF_18_ (*E*° = –1.61 V *vs.* Cp_2_Fe^0/+^).^45^

**Fig. 2:**
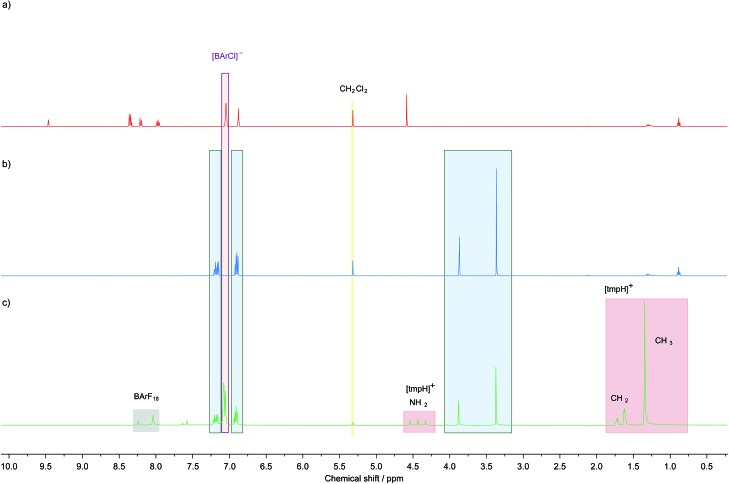
^1^H NMR spectra demonstrating the ability of [μ-H(BArF_18_)_2_]^–^ and [H-BArF_18_]^–^ to transfer hydride to **1**[BArCl] in CD_2_Cl_2_, (a) **1**[BArCl], (b) authentic **1**-H, and (c) an equimolar mixture of **1**[BArCl] and [tmpH][μ-H(BArF_18_)_2_] after 30 minutes at 20 °C.

Given that **1^+^** has a higher hydride ion affinity than B(C_6_F_5_)_3_,^38^ which has a greater hydride ion affinity than BArF_18_, one would expect salts of **1^+^** to abstract hydride from [(μ-H) (BArF_18_)_2_]^–^. This was confirmed experimentally by the formation of **1**-H when **1**[BArCl] {[BArCl]^–^ = tetra(3,5-dichlorophenyl)borate} was treated with an equivalent of [tmpH][(μ-H) (BArF_18_)_2_] (Fig. 2c). This suggests that BArF_18_ is highly suitable as a hydride shuttle for carbon-based electrochemical–FLPs derived from **1**-H/**1^+^**, and may provide a means of overcoming the high kinetic barrier for H_2_ activation by FLPs comprised of this carbon-based Lewis acid alone.

**Fig. 3:**
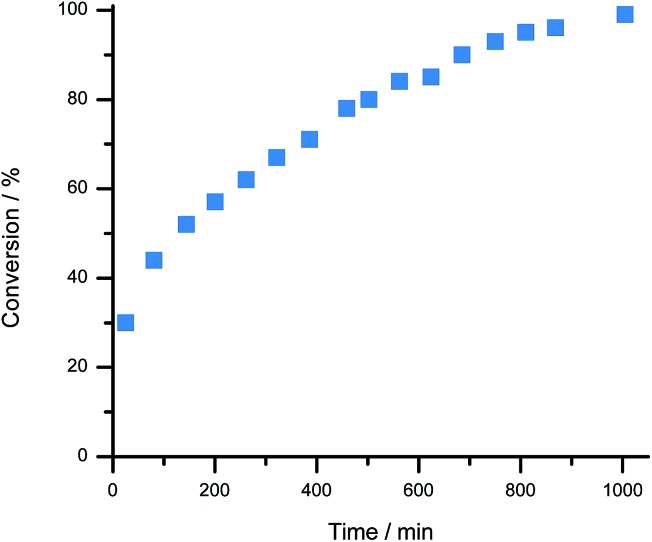
Progress of H_2_ activation by **1**[BArCl] in the presence of the mediator, BArF_18_. H_2_ (4 bar) was admitted to a sample of **1**[BArCl] (0.028 mmol, 1.0 equivalents), BArF_18_ (0.064 mmol, 2.3 equivalents), and 2,6-lutidine (0.048 mmol, 1.7 equivalents) in CD_2_Cl_2_ and the formation of **1**-H product was monitored using ^1^H NMR spectroscopy.

For proof of concept, a sample of **1**[BArCl] (1.0 equivalent), BArF_18_ (2.3 equivalents) and 2,6-lutidine (1.7 equivalents) in CD_2_Cl_2_ were combined. 2,6-Lutidine was chosen as the Lewis base because it is known to be compatible with **1^+^** and allows direct comparison to previous work.^38^ Importantly, in a control experiment 2,6-lutidine was found to be compatible with BArF_18_ as a FLP, with no evidence for adduct formation observed by NMR spectroscopy when an equimolar mixture of BArF_18_ and 2,6-lutidine was left for 2 days in CD_2_Cl_2_ (Fig. S8–S10†). On exposure of the three component mixture to H_2_ (4 bar) at room temperature, the progress of **1**-H formation was monitored by the disappearance of the CH signal of **1^+^** (at *δ* 9.4 ppm) and the appearance of the CH_2_ signal of **1**-H (at *δ* 3.9 ppm) in the ^1^H NMR spectrum (Fig. 3 and S4†). After only 25 minutes at 20 °C, 30% of **1**[BArCl] had been converted to **1**-H; quantitative conversion was achieved after 17 hours. This represents a significant improvement in H_2_ cleavage rate compared to **1^+^**/lut in the absence of BArF_18_, which requires over 9 days of heating at 60 °C before it approaches completion.^38^ Additionally, no evidence for CO binding was observed *via* NMR spectroscopy when the three component mixture (**1^+^**/BArF_18_/lut) was sparged with pure CO gas for 30 seconds (Fig. S5 and S7b†). On admission of excess H_2_ to the sample headspace, the usual formation of **1**-H occurred with no discernible signals corresponding to a formyl-borate species (Fig. S7†).^48^ This suggests that, in contrast to Pt or bioinspired organometallic electrocatalysts for H_2_ oxidation,^5^,^14^ our electrochemical–FLPs are CO tolerant and are not poisoned or otherwise inhibited, even in the presence of significant CO.

### Electrochemical–FLP experiments

**Fig. 4:**
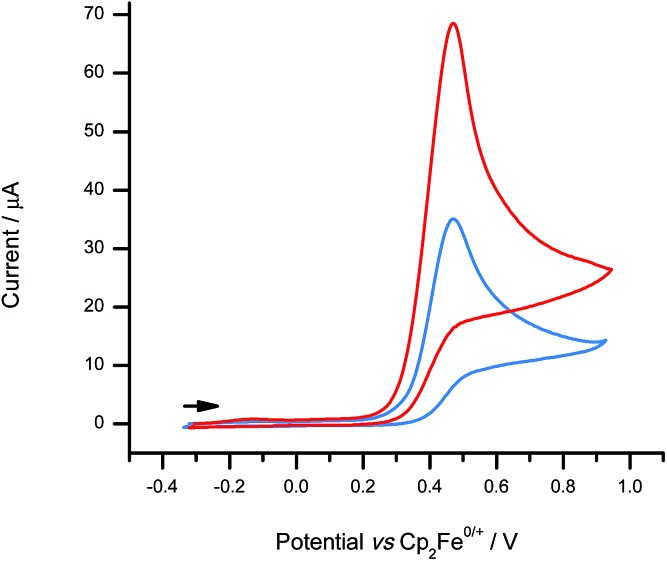
CVs comparing the electrochemical behaviour of **1**-H (1.8 mM) with (red line) and without (blue line) the addition of excess Brønsted base (2,6-lutidine; 6.7 mM) at a scan rate of 100 mV s^–1^.

Cyclic voltammetry was performed at a glassy carbon electrode (GCE) on solutions of **1**-H in CH_2_Cl_2_ containing 0.1 M [^*n*^Bu_4_N][B(C_6_F_5_)_4_] as a weakly-coordinating supporting electrolyte. In the absence of Brønsted base, cyclic voltammograms (CVs) of **1**-H exhibit a single-electron oxidation wave that is devoid of a back-peak (appears to be irreversible) until scan rates exceed 300 mV s^–1^ (Fig. S11†). In the presence of excess 2,6-lutidine, electrochemical reversibility is lost at all scan rates (Fig. S12†) and the peak current obtained for **1**-H approximately doubles (Fig. 4) – a 2-fold increase in peak current is observed at 50 mV s^–1^ and a 1.7-fold increase is observed at 2000 mV s^–1^. This effect is highly indicative of an underlying ECE-DISP1 mechanism, as reported by Savéant and co-workers previously.^43^ A peak potential of +0.47 V *vs.* Cp_2_Fe^0/+^ was obtained for **1**-H at the 100 mV s^–1^ scan rate. This is represents a 1 V decrease in the potential that is required for H_2_ oxidation at a GCE, a very significant energy saving that is equivalent to *ca.* 197 kJ mol^–1^, and provides a further 110 mV improvement over the previous most suitable borenium-based electrochemical–FLP system.^35^

Note that 2,6-lutidine has the added benefit of being electrochemically inactive within the potential window of our electrolyte system. This is unlike the phosphine and aliphatic amine bases used in our previous electrochemical–FLP studies which oxidize at similar potentials to the borohydrides, leading to electrode passivation and failure of the system.

### Applied H_2_ oxidation and electrocatalyst recyclability

The **1^+^**/BArF_18_/lut system was next applied towards the *in situ* oxidation of H_2_ with the intention of investigating whether the electrocatalyst (**1^+^**) could participate in successive charging and discharging cycles. The advantage of using BArF_18_ as a hydride shuttle is that the oxidation potential of [(μ-H) (BArF_18_)_2_]^–^ is on the limit of the oxidative potential window, and does not interfere with the measurement of **1**-H concentration at the electrode surface.

**Fig. 5:**
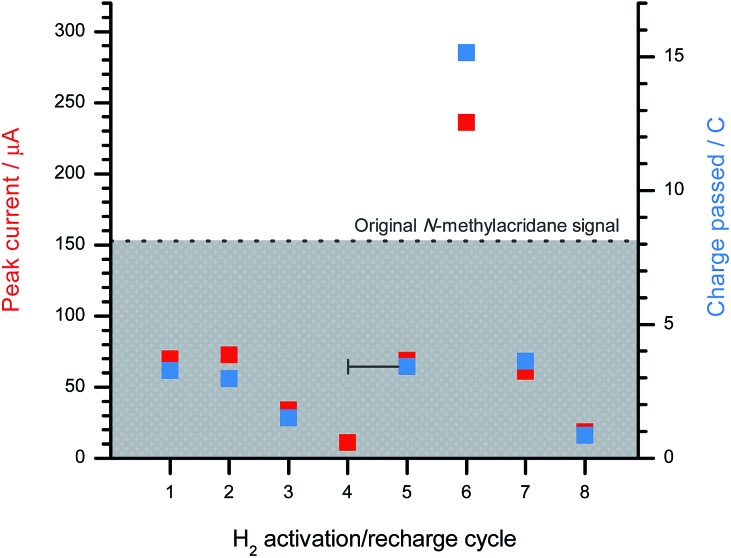
The peak current obtained at a GCE (left *y*-axis, red) and the charge passed at a Toray carbon paper electrode (right *y*-axis, blue) after sparging a freshly generated **1**[B(C_6_F_5_)_4_] (3.7 mM, 10 mol%) solution in CH_2_Cl_2_ with H_2_ for 20 minutes in the presence of 2,6-lutidine (41 mM, 11 equivalents) and BArF_18_ (37 mM, 1 equivalent). The dotted line represents the peak current/charge passed for the original **1**-H sample, which was converted to **1**[B(C_6_F_5_)_4_] *via* bulk electrolysis, and provides a reference point for the following H_2_ activation cycles. Additional 2,6-lutidine was added from cycle 4 onwards.

A sample of **1**[B(C_6_F_5_)_4_] was electrosynthesised *via* the controlled-potential bulk electrolysis of **1**-H (0.1 equivalent) in the presence of excess 2,6-lutidine (11 equivalents) at a Toray carbon paper electrode. An initial CV scan of **1**-H (recorded at a GCE) produced a peak current of 153 μA, and 8.03C of charge was passed during the initial bulk electrolysis step – this data is represented by the dotted line in Fig. 5. The formation of **1^+^** was further indicated by the solution turning bright yellow.

An equivalent of BArF_18_ was added (relative to the catalyst, **1^+^**, which is present at 10 mol%) and the sample was sparged with H_2_ gas for 20 minutes before a CV was recorded at the GCE. The CV clearly demonstrated the regeneration of considerable amounts of **1**-H, even at this short sparging time, with the peak current for this first H_2_ activation cycle at 46% (in agreement with the NMR studies above) of that passed for the original **1**-H sample prior to bulk electrolysis. The sample was electrolyzed back to **1**[B(C_6_F_5_)_4_], passing 3.28C of charge (41% of that passed for the original sample). H_2_ activation was then repeated for the second time (again, with only a 20 minute sparge) at which point the observed peak current was comparable to that obtained for the first H_2_ activation. On repeating the H_2_ activation a third time, the peak current and charge passed for **1**-H during bulk electrolysis was somewhat diminished compared to the initial two attempts (to *ca.* 20% of the original sample values). A fourth H_2_ activation attempt was unsuccessful, with no regeneration of **1**-H.

It was suspected that the system was no longer turning over due to the depletion of 2,6-lutidine *via* its sequestration by protons generated during the bulk electrolysis of **1**-H and also in the H_2_ activation cycles by the FLP. In a fuel cell, H_2_ oxidation constitutes only one half-reaction of the redox couple; the other half-reaction, O_2_ reduction, would consume any protons that are generated during H_2_ oxidation and regenerate the Brønsted base. Thus, at this point in the experiment, the number of regeneration cycles was limited by the quantity of available 2,6-lutidine. To overcome this issue, an additional 10 equivalents of 2,6-lutidine were added to the sample, which was then subjected to a further 20 minute sparge with H_2_. Reassuringly, this fifth H_2_ activation run successfully regenerated **1**-H in similar concentrations (*ca.* 45% of the original sample concentration after a 20 minute sparge) to those obtained during the first two H_2_ activation attempts. The sample was then subjected to bulk electrolysis.

To investigate the effect of exposing the sample to H_2_ for longer periods of time, the sample was left sealed under H_2_ for 2.5 days. To great surprise, the resulting CV (cycle 6) exhibited a 1.5-fold increase in peak current compared to the original **1**-H sample. It is likely that excess [lutH][(μ-H) (BArF_18_)_2_] builds up in solution once all **1^+^** (present at 10 mol% *cf.* the borane) has been converted back to **1**-H. As **1**-H undergoes oxidation at the electrode surface, the electrogenerated **1^+^** is rapidly converted back to **1**-H *via* reaction with the excess [(μ-H) (BArF_18_)_2_]^–^. This leads to an enhancement in the peak current of the **1**-H oxidation wave *i.e.* a perceived electrocatalytic effect. This effect was confirmed experimentally by treating a sample of **1**-H with increasing quantities (0, 0.5, 1, and 2 equivalents) of the hydride donor [^*n*^Bu_4_N][HB(C_6_F_5_)_3_] (Fig. S13a†). The addition of [^*n*^Bu_4_N][HB(C_6_F_5_)_3_] resulted in a proportional increase in the peak current of the **1**-H wave (Fig. S13b†). Note that whilst [HB(C_6_F_5_)_3_]^–^ is redox active, its peak potential is observed at +0.88 V *vs.* Cp_2_Fe^0/+^ and therefore does not interfere with the **1**-H oxidation wave. The fact that the peak current of **1**-H increases, with no observable wave corresponding to [HB(C_6_F_5_)_3_]^–^, suggests that hydride shuttling occurs within the timescale of the electrode process – *i.e.* the system is not only rechargeable, but it is catalytic and turning over many times per H_2_-charge cycle. Digital simulation of this electrochemical data determined the turnover frequency of the hydride shuttling process to be 2.7 ± 0.2 × 10^4^ s^–1^.

Henceforth, excess 2,6-lutidine (10 equivalents) was added after each bulk electrolysis step to ensure that that system recyclability was not limited by the concentration of Brønsted base. Following bulk electrolysis, the sample containing **1^+^** was subjected to further H_2_ activation (recharging, cycle 7) and bulk electrolysis cycles (discharging) until **1**-H could no longer be regenerated. Only one successful regeneration cycle was performed before no further **1**-H formation was observed. Since the 2,6-lutidine concentration was not the limiting factor, it is likely that the deactivation of the electrocatalytic system resulted from the decomposition of the boron-based Lewis acid, BArF_18_ (whose concentration was not altered from the initial experiment in the series), over the course of several charging and discharging cycles. Indeed, BArF_18_ is relatively sensitive to trace amounts of adventitious air and moisture. Despite this, the **1^+^**/**1**-H carbon-based Lewis acid system was confirmed to still be fully active when the addition of [^*n*^Bu_4_N][HB(C_6_F_5_)_3_] resulted in successful recovery of the oxidation wave corresponding to **1**-H.

## Conclusions

The **1^+^**/BArF_18_/lut system provides a new and improved electrochemical–FLP approach to H_2_ oxidation by combining the best attributes of two different Lewis acids: one carbon-based with excellent electrochemical attributes, and one boron-based with excellent H_2_ activating attributes as part of a FLP. Unlike conventional, precious metal or biomimetic electrocatalysts, this system is highly tolerant to CO. The pre-activation of H_2_ in the form of **1**-H results in an astonishing 1 V decrease in the potential that is required for H_2_ oxidation at ubiquitous carbon electrodes. This represents a significant decrease in the required energetic driving force for H_2_ oxidation (equivalent to *ca.* 197 kJ mol^–1^) and a further 110 mV improvement over previous electrochemical–FLP systems. In addition to this (and in contrast to our previous electrochemical–FLP systems) there are no H_2_ evolution side-reactions due to the reaction of incoming hydride with electrogenerated H^+^; this leads to a marked improvement in efficiency and recyclability.

The completely metal-free system is electrocatalytic with respect to the carbon-based Lewis acid **1^+^** and can be turned over multiple times without any loss of activity. The “hydride shuttle” effect provided by the synergistic interaction of BArF_18_ and **1^+^** gives rise to a significant improvement in the overall rates of H_2_ cleavage and the generation of **1**-H by the carbon-based FLP.

We see two routes to further improve this electrochemical–FLP system. One, to develop a boron-based FLP that exhibits a greater stability to air and moisture whilst retaining the ability to rapidly cleave H_2_ and to function as an efficient “hydride shuttle”. Indeed, we have already demonstrated that solutions of B(C_6_F_5_)_3_ in 1,4-dioxane can be rendered water tolerant simply by operating at increased pressures of H_2_.^33^ Alternatively, an analogous carbon-based Lewis acid is required that is capable of rapid H_2_ activation when combined with a suitable Lewis base, without requiring the presence of any additional boron-based Lewis acid as a hydride shuttle. Both approaches form part of our ongoing research efforts.

## Supplementary Material

Supplementary information
