# Supplementary material for: Metal-free electrocatalytic hydrogen oxidation using frustrated Lewis pairs and carbon-based Lewis acids
Source: Chem Sci. 2016 Jan 6;7(4):2537–43. doi: 10.1039/c5sc04564a (PMC5477046; doi:10.1039/c5sc04564a)
Supplement: Supplementary file 1 [file SC-007-C5SC04564A-s001.pdf]

Electronic Supplementary Material (ESI) for Chemical Science.
This journal is © The Royal Society of Chemistry 2016

Supplementary Information

Metal-free electrocatalytic hydrogen oxidation using frustrated Lewis pairs and carbon-based Lewis acids

Elliot J. Lawrence,[a] Ewan R. Clark,[b] Liam D. Curless,[b] James M. Courtney,[a] Robin J. Blagg,[a] Michael J. Ingleson,*[b] Gregory G. Wildgoose*[b]

[a]Energy & Materials Laboratory, School of Chemistry, University of East Anglia, Norwich Research Park, Norwich, NR4 7TJ, United Kingdom.

[b]School of Chemistry, University of Manchester, Oxford Road, Manchester M13 9PL, United Kingdom.

Contents

S1. Experimental details 2

S2. NMR spectra 3

S3. Electrochemical data 13

S4. References 15

S1. Experimental details

All synthetic reactions and manipulations were performed under a rigorously dry N_2 atmosphere using standard Schlenk-line techniques on a dual manifold vacuum/inert gas line or an MBraun glovebox. Anhydrous solvents were dried by reflux over appropriate drying agents and were collected by distillation. All solvents were sparged with nitrogen gas to remove any trace of dissolved oxygen and stored in ampoules over activated 4 Å molecular sieves. H_2 gas (99.995 %) was purchased from BOC gases and was used without further purification. **1**-H, **1**[BArCl];[S1] $BArF_{18}$,[S2] [tmpH][(μ-H)($BArF_{18}$)$_2$];[S3,S4] [^{n}Bu$_4$N][HB(C_6F_5)$_3$],[S5] and [^{n}Bu$_4$N][B(C_6F_5)$_4$][S6,S7] were prepared according to the cited literature methods.

NMR spectra were recorded using a Bruker Avance DPX-500 MHz spectrometer. Chemical shifts are reported in ppm and are referenced relative to appropriate standards: ^{19}F ($CFCl_3$); ^{11}B ($Et_2O{\cdot}BF_3$).

All electrochemical experiments were performed in an MBraun glovebox using either an Autolab PGSTAT 30 or PGSTAT 302N computer-controlled potentiostat (Utrecht, The Netherlands). All electrochemical samples were prepared in CH_2Cl_2 containing 0.1 M [^{n}Bu$_4$N][B(C_6F_5)$_4$] as a weakly coordinating electrolyte salt.

Cyclic voltammetry (CV) was performed using a three-electrode configuration consisting of a glassy carbon macrodisk working electrode (GCE) (diameter of 3 mm; BASi, Indiana, USA) combined with a Pt wire counter electrode (99.99%; GoodFellow, Cambridge, UK) and a Ag wire *pseudo*-reference electrode (99.99%; GoodFellow, Cambridge, UK).

Controlled-potential bulk electrolysis was performed using a three-electrode configuration consisting of a wet-proofed Toray carbon paper 060 (20 wt% PTFE treatment; Fuel Cell Store, Texas, USA) working electrode combined with a Ag wire *pseudo*-reference electrode (99.99%, GoodFellow, Cambridge, UK) and a Pt gauze counter electrode (52 mesh woven from 0.1 mm diameter wire, 99.9%; Alfa Aesar, Massachusetts, USA). The working and *pseudo*-reference electrodes were separated from the counter electrode compartment by a microporous ceramic frit (4 Å pore size).

The Ag wire *pseudo*-reference electrodes were calibrated to the $Cp_2Fe^{0/+}$ couple in CH_2Cl_2 at the end of each run to allow for any drift in potential, following IUPAC recommendations.[S8]

S2. NMR spectra

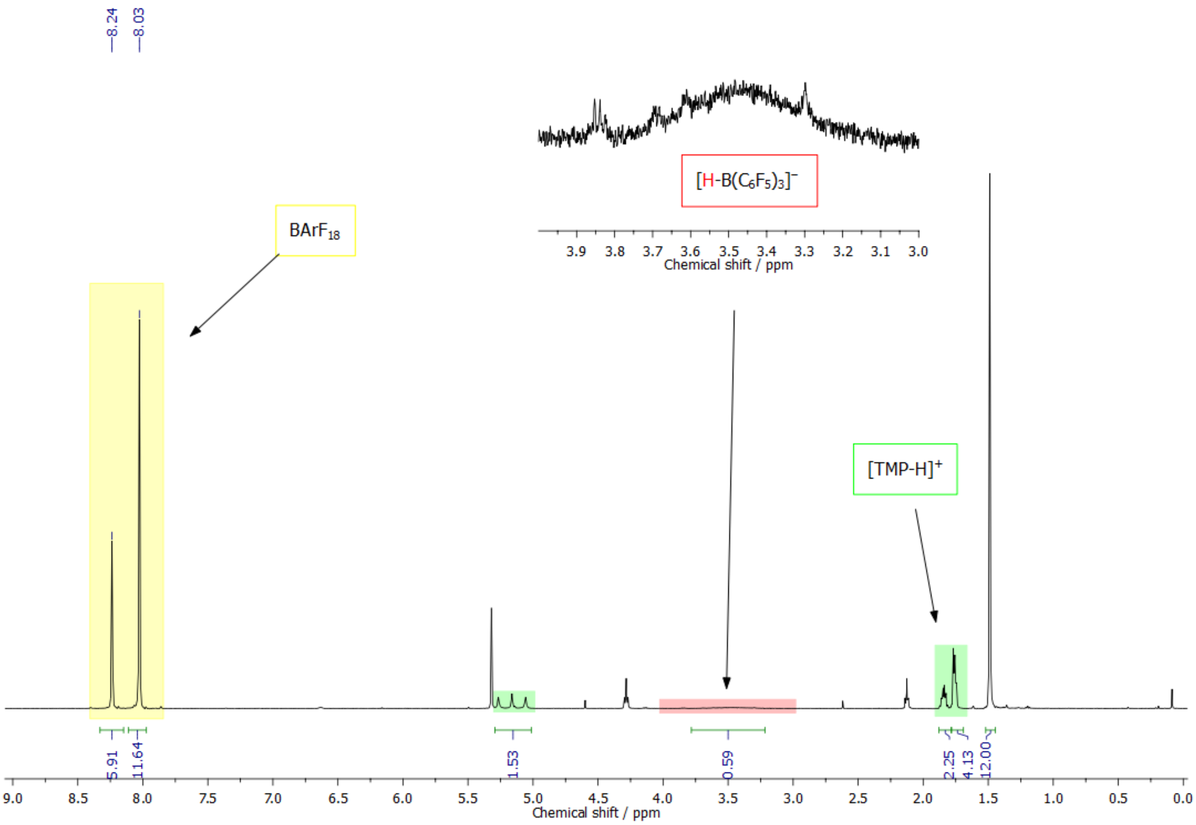

Fig. S1 ^{1}H NMR spectrum of a mixture of [tmpH][μ-H($BArF_{18}$)$_2$] and $B(C_6F_5)_3$ in CD_2Cl_2; the ability of [tmpH][μ-H($BArF_{18}$)$_2$] as a hydride donor is demonstrated by the generation of $[HB(C_6F_5)_3]^-$ when treated with excess $B(C_6F_5)_3$.

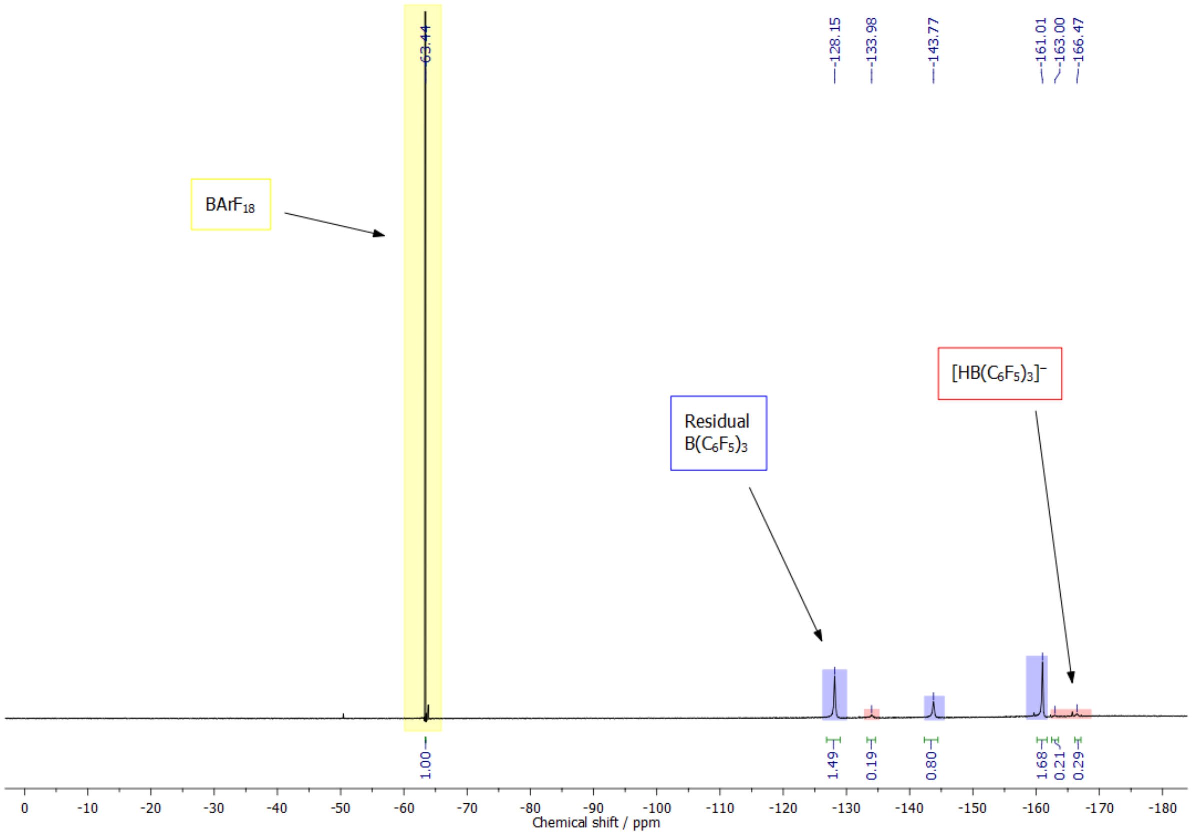

Fig. S2 $^{19}F\{^{1}H\}$ NMR spectrum of a mixture of [tmpH][μ-H($BArF_{18}$)$_2$] and $B(C_6F_5)_3$ in CD_2Cl_2; the ability of [tmpH][μ-H($BArF_{18}$)$_2$] as a hydride donor is demonstrated by the generation of $[HB(C_6F_5)_3]^-$ when treated with excess $B(C_6F_5)_3$.

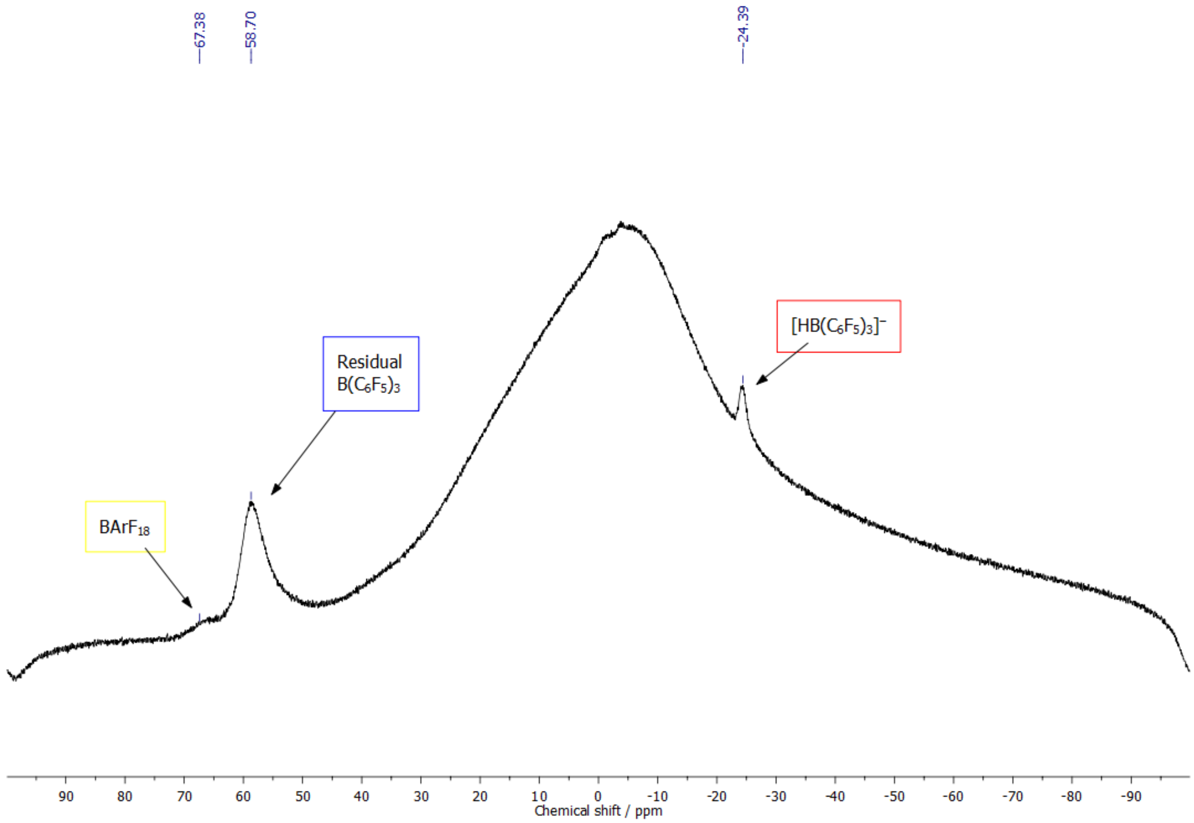

Fig. S3 ^{11}B NMR spectrum of a mixture of [tmpH][μ-H(BArF$_{18}$)$_2$] and B(C_6F_5)$_3$ in CD_2Cl_2; the ability of [tmpH][μ-H(BArF$_{18}$)$_2$] as a hydride donor is demonstrated by the generation of [HB(C_6F_5)$_3$]$^-$ when treated with excess B(C_6F_5)$_3$.

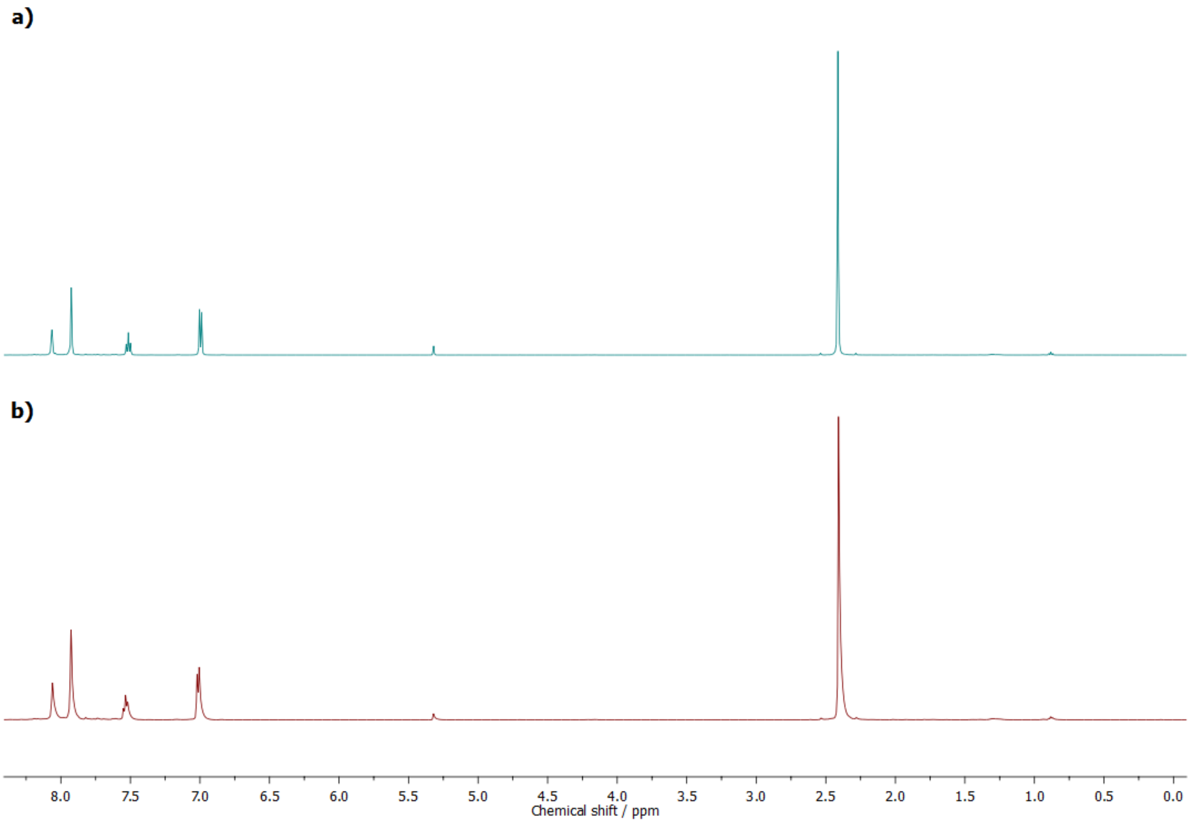

Fig. S4 ^1H NMR spectroscopy demonstrates that $BArF_{18}$ (0.08 mmol) and 2,6-lutidine (0.08 mmol) remain as individual components after 2 days in CD_2Cl_2 solution. (a) Freshly prepared sample. (b) Sample after 2 days.

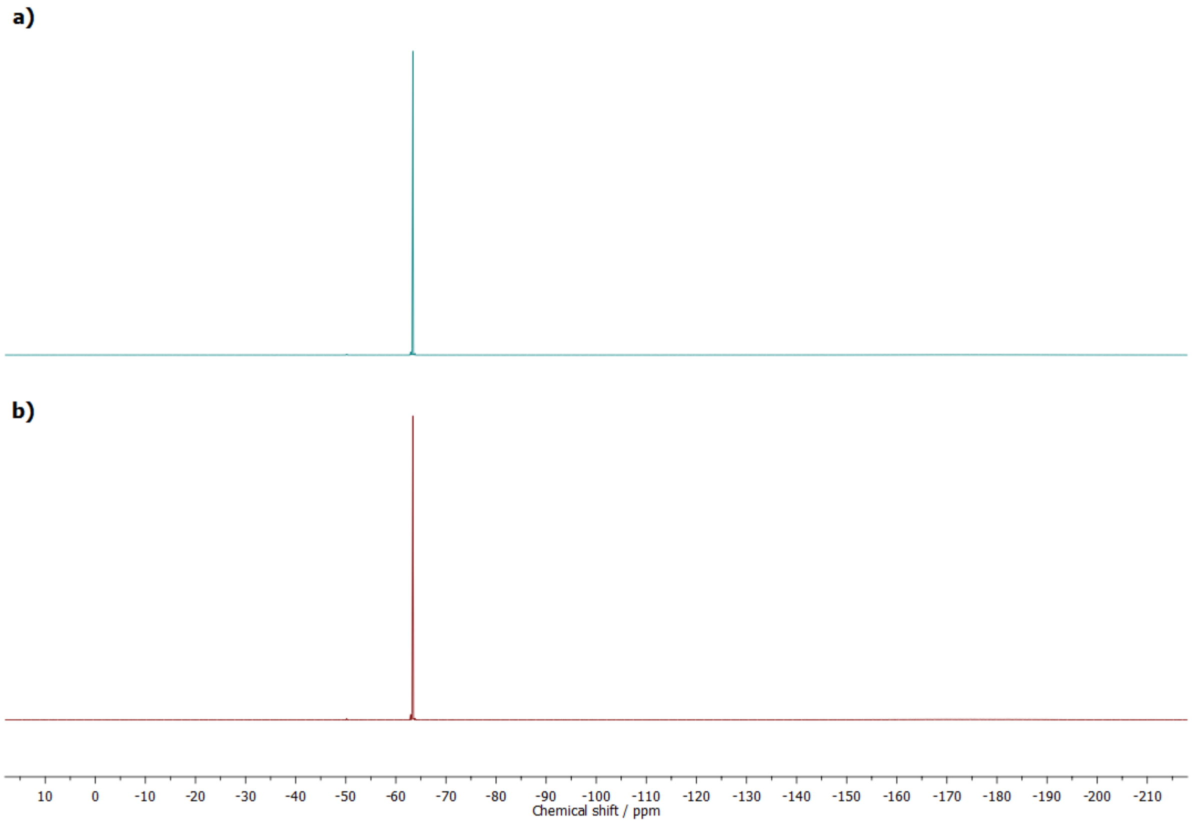

Fig. S5 $^{19}F\{^1H\}$ NMR spectroscopy demonstrates that $BArF_{18}$ (0.08 mmol) and 2,6-lutidine (0.08 mmol) remain as individual components after 2 days in CD_2Cl_2 solution. (a) Freshly prepared sample. (b) Sample after 2 days.

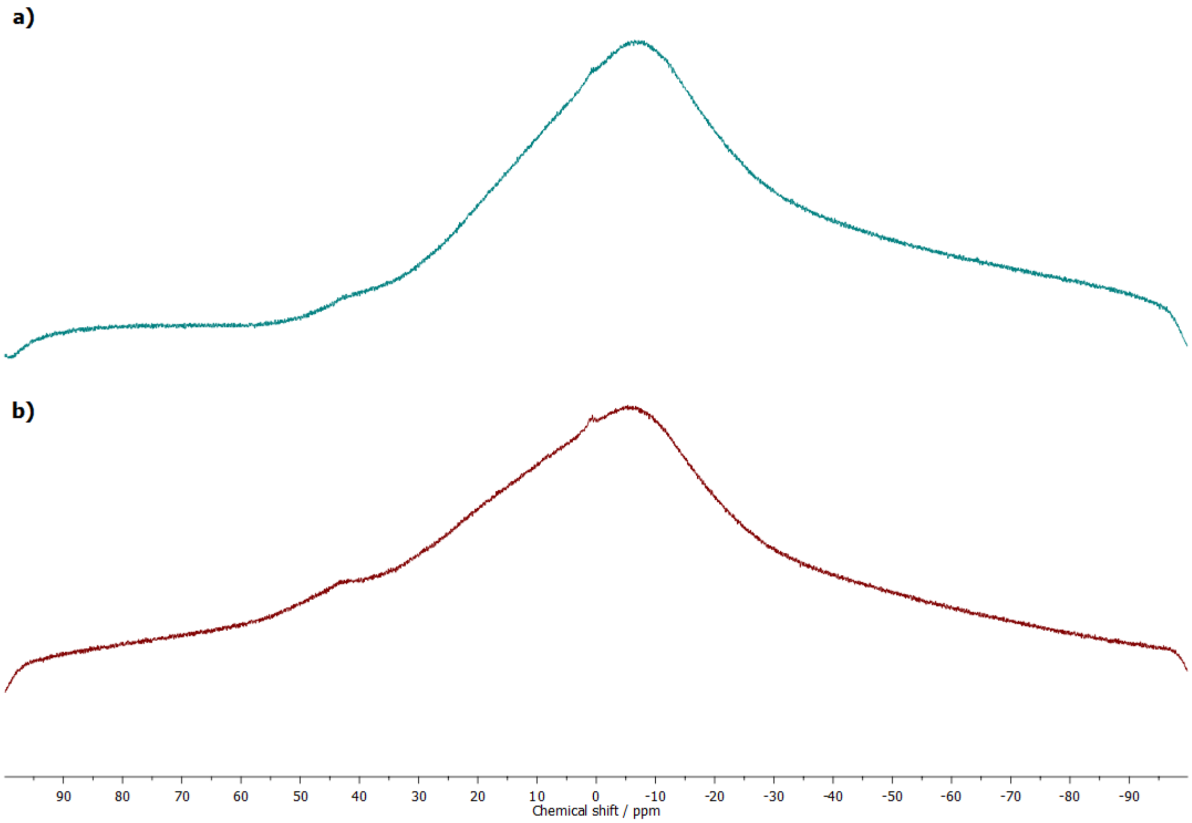

Fig. S6 ^{11}B NMR spectroscopy demonstrates that $BArF_{18}$ (0.08 mmol) and 2,6-lutidine (0.08 mmol) remain as individual components after 2 days in CD_2Cl_2 solution. (a) Freshly prepared sample. (b) Sample after 2 days.

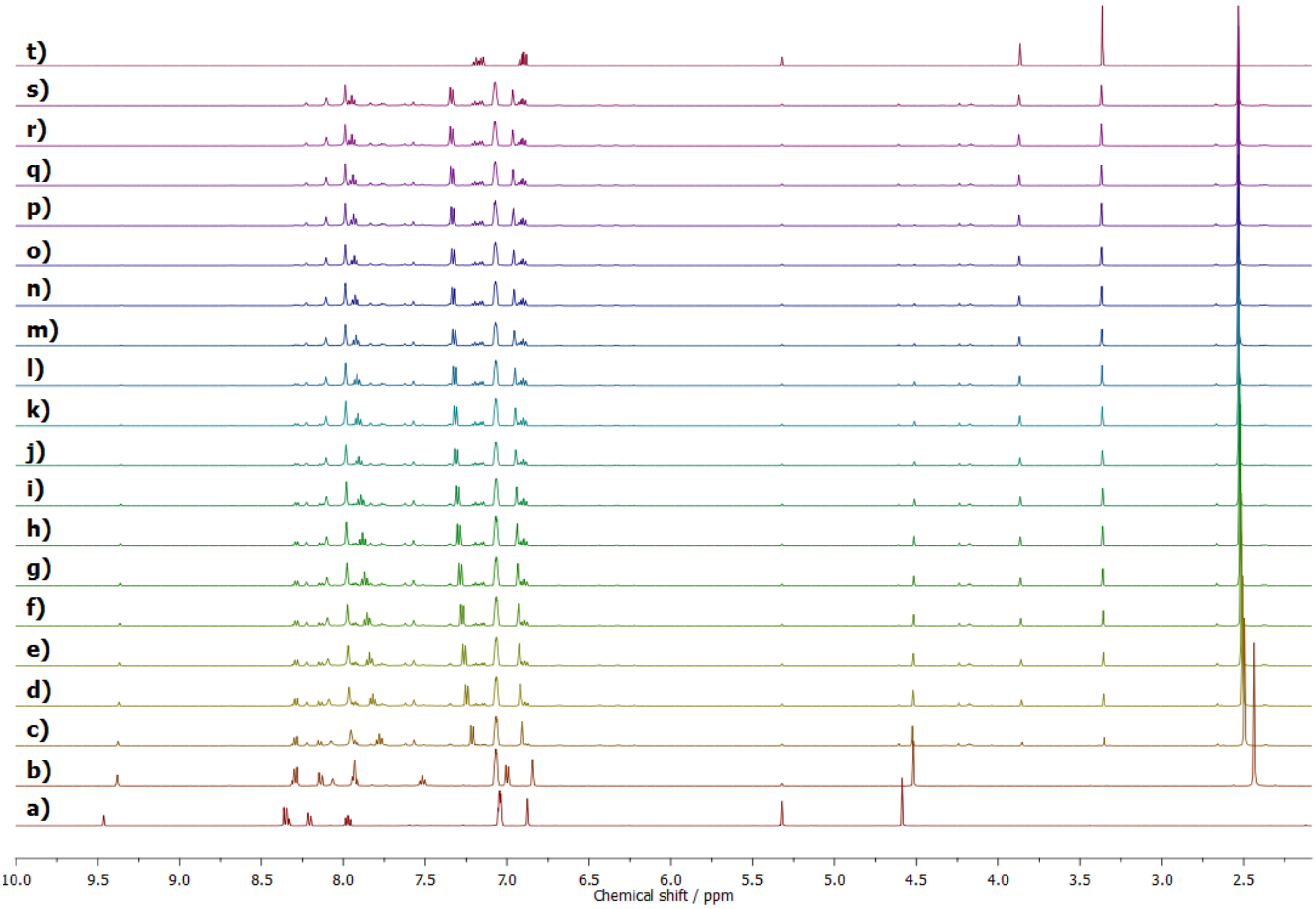

Fig. S7 ^{1}H NMR spectra showing the progress of H_2 activation by the **1**[BArCl]/2,6-lutidine system (in CD_2Cl_2) in the presence of $BArF_{18}$. (a) Authentic **1**[BArCl]. (b) Sample of **1**[BArCl] (1.0 eq.), $BArF_{18}$ (2.3 eq.), and 2,6-lutidine (1.7 eq.) under N_2. (c-s) Monitoring the progress of **1**-H formation following the admission of H_2 (4 bar) to the sample in b (after 0.5-17 h). (t) Authentic **1**-H.

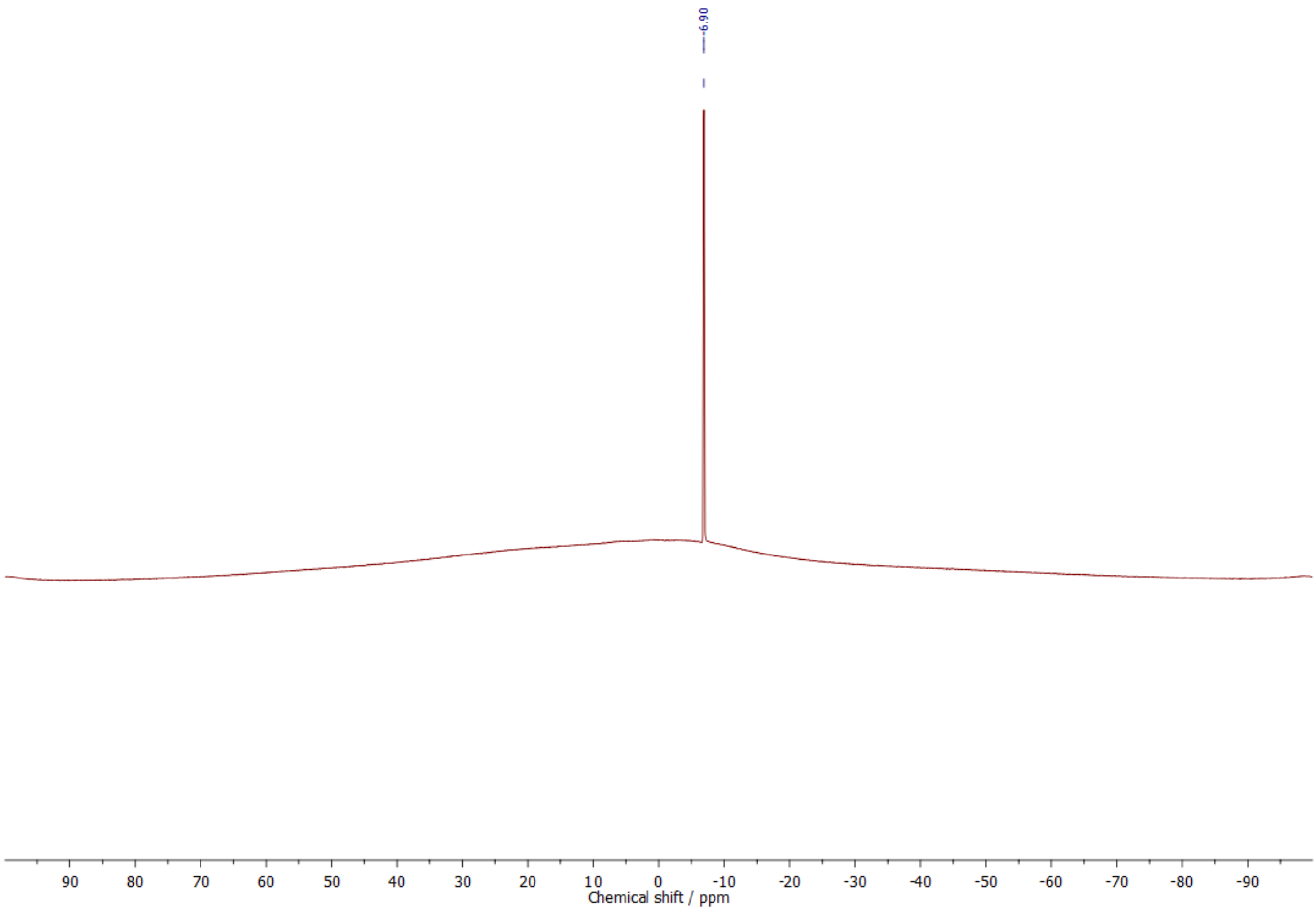

Fig. S8 ^{11}B NMR spectra of the **1**[BArCl] (1.0 eq.)/$BArF_{18}$ (2.3 eq.)/2,6-lutidine (2.0 eq.) system (in CD_2Cl_2) after a 30 second sparge with pure CO gas demonstrates that the system is tolerant towards CO.

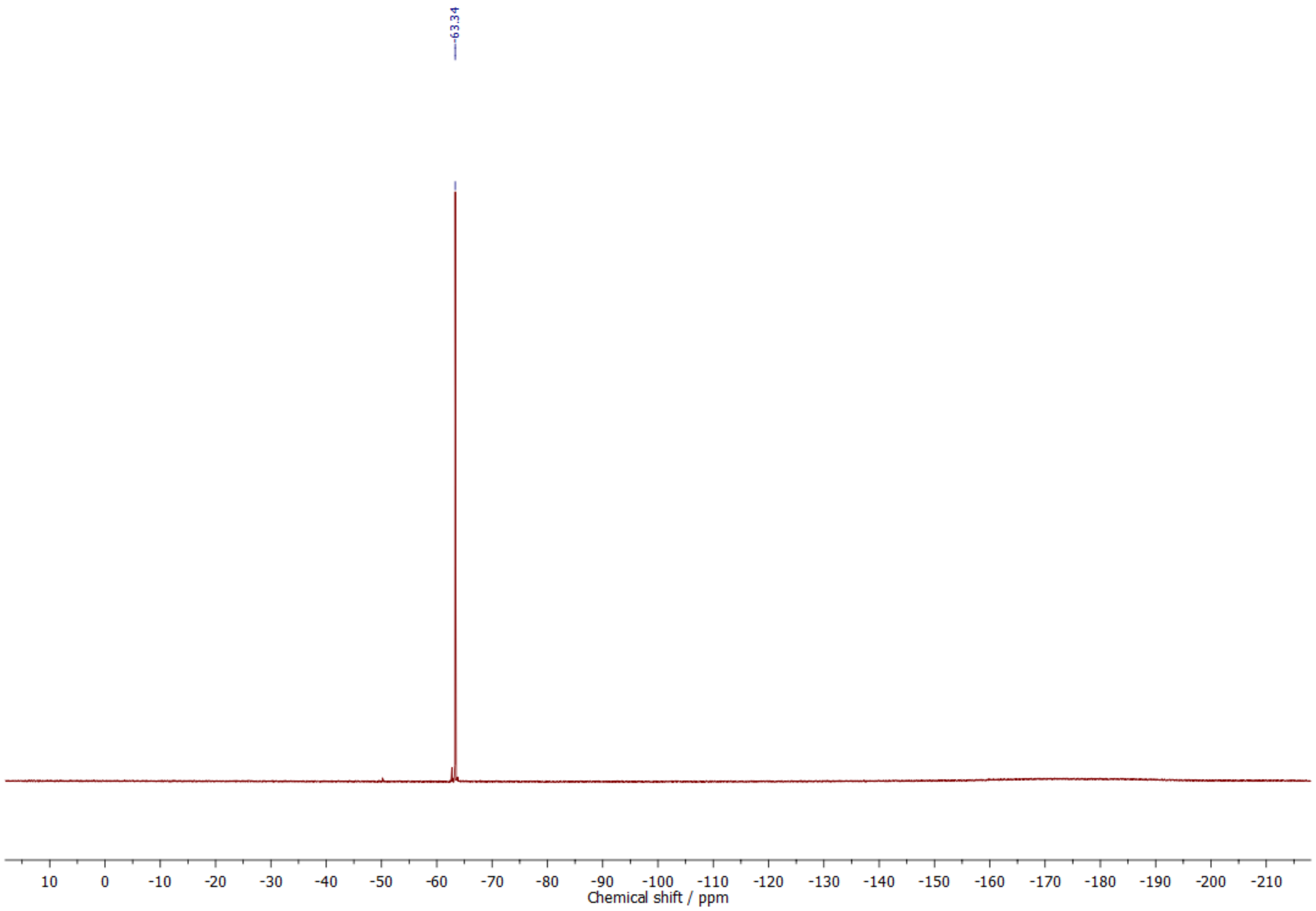

Fig. S9 $^{19}F\{^1H\}$ NMR spectra of the **1**[BArCl] (1.0 eq.)/$BArF_{18}$ (2.3 eq.)/2,6-lutidine (2.0 eq.) system (in CD_2Cl_2) after a 30 second sparge with pure CO gas demonstrates that the system is tolerant towards CO.

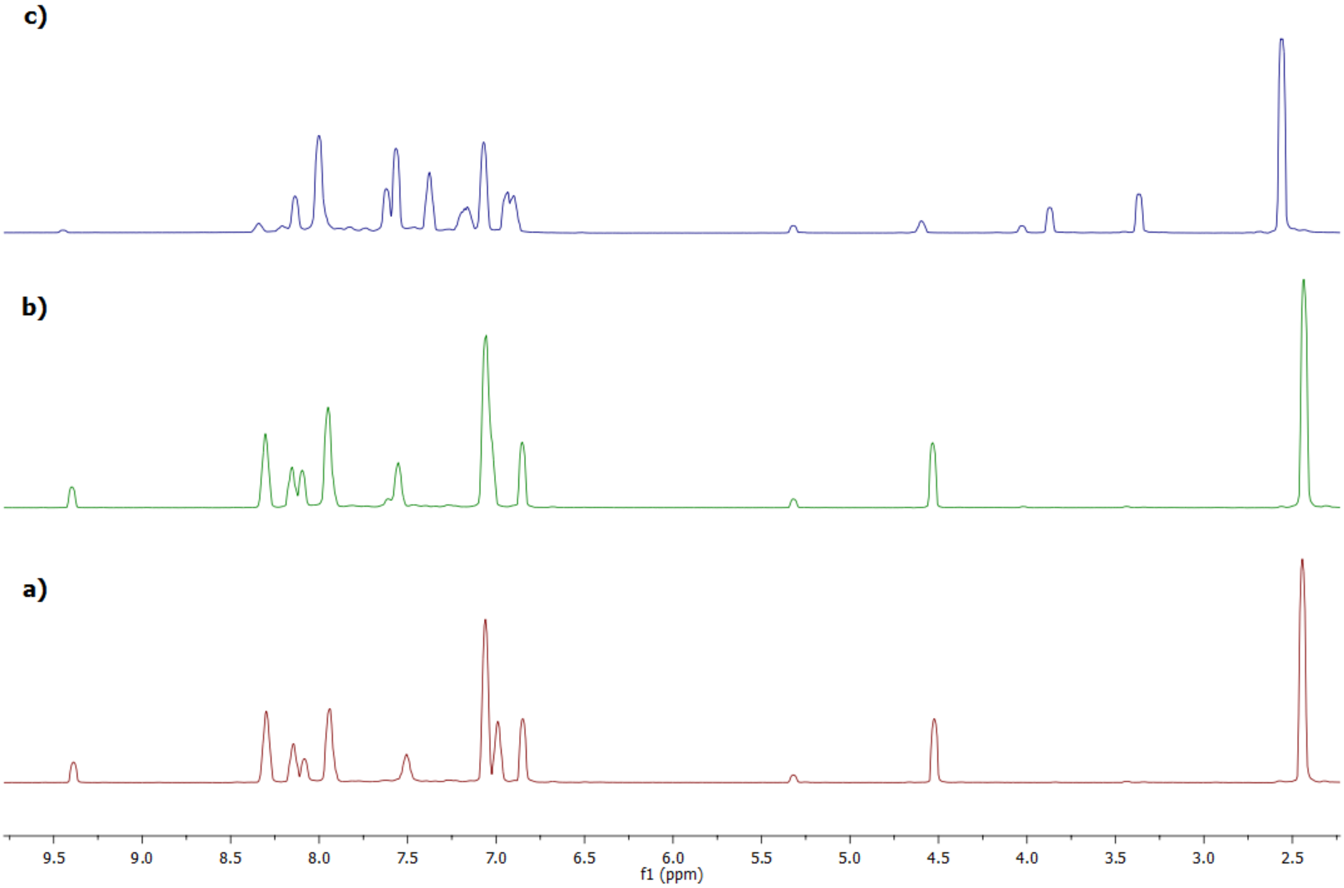

Fig. S10 ^{1}H NMR spectra of a sample of **1**[BArCl] (1.0 eq.), $BArF_{18}$ (2.3 eq.), and 2,6-lutidine (2.0 eq.) in CD_2Cl_2 (a) under N_2, (b) after a 30 second sparge with pure CO gas, and (c) after a 30 second sparge with pure CO gas and exposure to excess H_2 over 24 hours.

S3. Electrochemical data

**1**-H	⇌	[**1**-H]$^{•+}$ + e^-	
[**1**-H]$^{•+}$ + B	⇌	**1**$^•$ + [B-H]$^+$	
**1**$^•$	⇌	**1**$^+$ + e^-	*ECE*
**1**$^•$ **+ [1**-H]$^{•+}$	→	**1**$^+$ + **1**-H	*DISP*
**1**-H	→	**1**$^+$ + 2e^- + H$^+$	*Overall reaction*

Scheme S1. ECE-DISP1 mechanism of **1**-H oxidation in the presence of Brønsted base (B). Single-electron oxidation of **1**-H is followed by the deprotonation of the resulting radical cation, [**1**-H]$^{•+}$, by the Brønsted base. This homogeneous deprotonation reaction is the rate-determining step and generates the neutral radical **1**$^•$, which is then oxidized either immediately at the electrode surface (ECE) or homogeneously in solution *via* a disproportionation reaction with [**1**-H]$^{•+}$ (DISP).[S9]

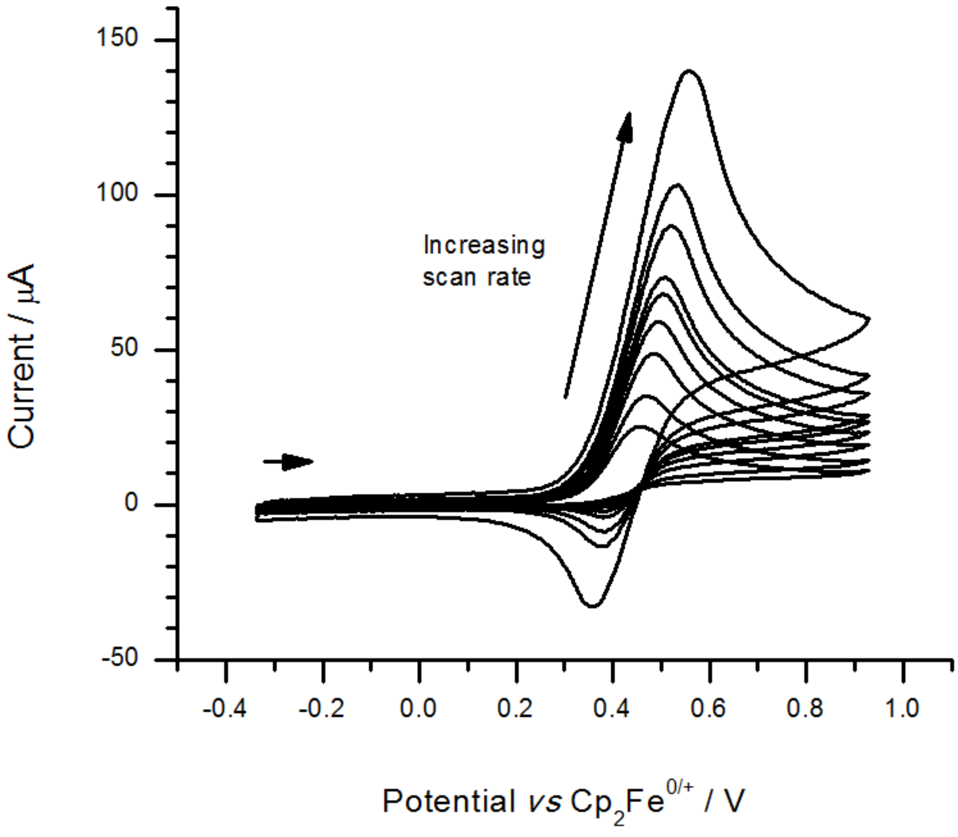

Fig. S11 CVs of **1**-H (1.8 mM) obtained in the absence of Brønsted base at scan rates of 50, 100, 200, 300, 400, 500, 750, 1000, and 2000 mV s^{-1}.

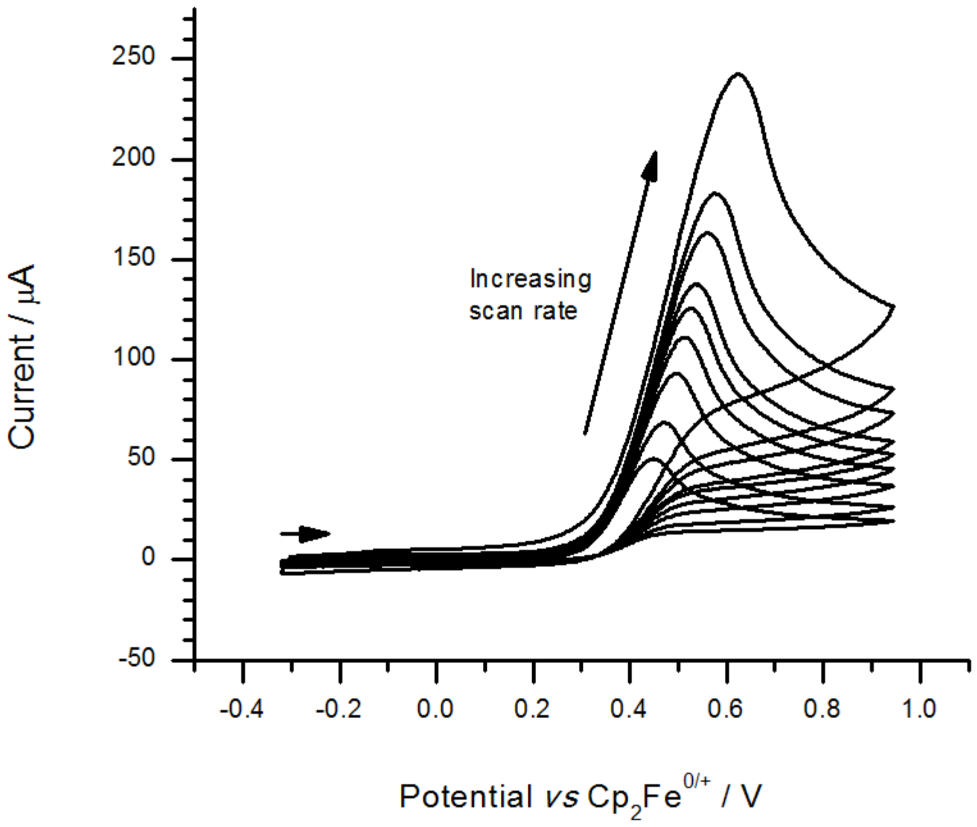

Fig. S12 CVs of **1**-H (1.8 mM) obtained in the presence of excess 2,6-lutidine (6.7 mM) at scan rates of 50, 100, 200, 300, 400, 500, 750, 1000, and 2000 mV s^{-1}.

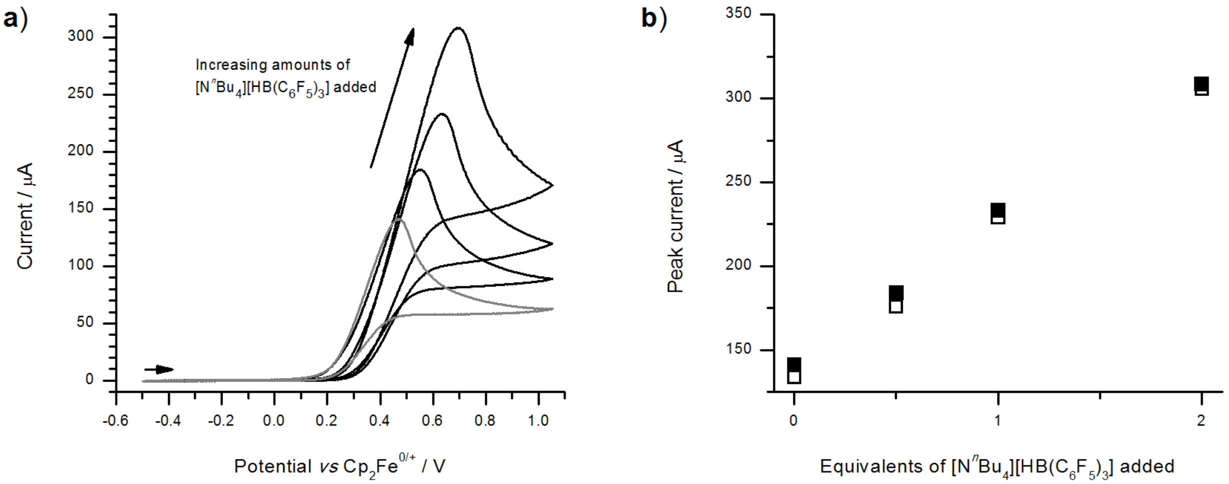

Fig. S13 (a) CVs showing the effect of adding increasing amounts of the hydride donor [^{n}Bu$_4$N][HB(C$_6$F$_5$)$_3$] (0.5, 1, and 2 equivalents, black lines) to a sample of **1**-H (4.1 mM, grey line) whilst in the presence of excess 2,6-lutidine (41 mM, 10 eq.). (b) Observed (closed squares) and simulated (open squares) peak current of the **1**-H signal with added [^{n}Bu$_4$N][HB(C$_6$F$_5$)$_3$].

S4. References

S1 E. R. Clark and M. J. Ingleson, *Angew. Chem. Int. Ed.*, 2014, **53**, 11306–11309.

S2 E. Lawrence, G. Wildgoose, A. Ashley, *ChemSpider SyntheticPages*, 2015, http://cssp.chemspider.com/805, DOI: 10.1039/SP805.

S3 T. J. Herrington, A. J. W. Thom, A. J. P. White and A. E. Ashley, *Dalton Trans.*, 2012, **41**, 9019.

S4 E. L. Kolychev, T. Bannenberg, M. Freytag, C. G. Daniliuc, P. G. Jones and M. Tamm, *Chem. Eur. J.*, 2012, **18**, 16938–16946.

S5 E. J. Lawrence, V. S. Oganesyan, D. L. Hughes, A. E. Ashley and G. G. Wildgoose, *J. Am. Chem. Soc.*, 2014, **136**, 6031–6036.

S6 E. Martin, D. L. Hughes and S. J. Lancaster, *Inorg. Chim. Acta*, 2010, **363**, 275–278.

S7 R. J. LeSuer, C. Buttolph and W. E. Geiger, *Anal. Chem.*, 2004, **76**, 6395–6401.

S8 G. Gritzner and J. Kůta, *Electrochim. Acta*, 1984, **29**, 869–873.

S9 P. Hapiot, J. Moiroux and J.-M. Savéant, *J. Am. Chem. Soc.*, 1990, **112**, 1337–1343.
